# Discrimination of Stressed and Non-Stressed Food-Related Bacteria Using Raman-Microspectroscopy

**DOI:** 10.3390/foods11101506

**Published:** 2022-05-22

**Authors:** Daniel Klein, René Breuch, Jessica Reinmüller, Carsten Engelhard, Peter Kaul

**Affiliations:** 1 Institute of Safety and Security Research, Bonn-Rhein-Sieg University of Applied Sciences, Von-Liebig-Straße 20, 53359 Rheinbach, Germany; rene.breuch@h-brs.de (R.B.); reinmuellerje@aol.com (J.R.); peter.kaul@h-brs.de (P.K.); 2 Department of Chemistry and Biology, University of Siegen, Adolf-Reichwein-Str. 2, 57076 Siegen, Germany; engelhard@chemie.uni-siegen.de; 3 Center of Micro- and Nanochemistry and Engineering, University of Siegen, Adolf-Reichwein-Str. 2, 57076 Siegen, Germany

**Keywords:** stress response, Raman-microspectroscopy, discriminant analysis, classification, bacteria

## Abstract

As the identification of microorganisms becomes more significant in industry, so does the utilization of microspectroscopy and the development of effective chemometric models for data analysis and classification. Since only microorganisms cultivated under laboratory conditions can be identified, but they are exposed to a variety of stress factors, such as temperature differences, there is a demand for a method that can take these stress factors and the associated reactions of the bacteria into account. Therefore, bacterial stress reactions to lifetime conditions (regular treatment, 25 °C, HCl, 2-propanol, NaOH) and sampling conditions (cold sampling, desiccation, heat drying) were induced to explore the effects on Raman spectra in order to improve the chemometric models. As a result, in this study nine food-relevant bacteria were exposed to seven stress conditions in addition to routine cultivation as a control. Spectral alterations in lipids, polysaccharides, nucleic acids, and proteins were observed when compared to normal growth circumstances without stresses. Regardless of the involvement of several stress factors and storage times, a model for differentiating the analyzed microorganisms from genus down to strain level was developed. Classification of the independent training dataset at genus and species level for *Escherichia coli* and at strain level for the other food relevant microorganisms showed a classification rate of 97.6%.

## 1. Introduction

The increasing demand for food, as well as the ever-increasing population of the planet makes the food sector an essential industry [1,2,3,4]. Due to the massive amount of 931 million tons of food waste, as well as the 600 million cases of food-borne illnesses occurring annually and the associated consumer fear, the research area of food safety is of particular interest [5,6,7]. In particular, bacterial detection is a critical concern in the food sector; for example, for determining shelf life dates [8,9]. Therefore, methods that identify the contamination of products at an early stage are of major importance [8,9].

However, the typical methods for detecting and determining bacteria, such as classical microbiological determination and immunological or genetic approaches are exceedingly expensive, difficult, and time-consuming due to the need of cultivation times, DNA extractions and well trained employees [9,10,11,12,13,14,15]. Another problem with these approaches is that they are standardized to laboratory conditions, resulting in the loss of real-world samples and the possibility of missing relevant information [10,16]. Because bacteria are typically exposed to a variety of external influences, both in the environment and directly on the samples to be examined, detection and determination methods must incorporate these external influences in order to account for rapid reactions of the microorganisms caused by external stress in their calculations [16,17,18,19]. This is especially essential because bacteria can not only adjust their metabolic activity in response to changing environments, but they can also change to a viable but non-culturable state, leaving them inaccessible to traditional determination methods [10,17,20,21]. Therefore, it is necessary to study the stress response of microorganisms and to consider it in the databases and classification models for quality control or shelf-life data determination [15,22].

These reactions can be studied by rapid and non-destructive vibrational spectroscopy on a microscopic level by coupling Raman or IR spectrometers with a microscopic system, as these couplings are able to study samples in the range of a few micrometers [1,23,24,25].

While Raman spectroscopy is based on the scattering of monochromatic light and the shift in polarizability, infrared (IR) spectroscopy is based on the absorption of polychromatic infrared light and the resulting change in dipole moment [23,25,26,27,28,29]. Certainly, IR-active vibrational modes often exhibit weak Raman signals and vice versa and so both methods provide complementary information about the molecular composition of microorganisms that thus contribute to classification [8,21,30,31].

IR spectroscopy is more susceptible to interference due to the materials used, the disturbance by water and the strong influence of sample thickness, as well as external influences, but it is still easier to handle [21,28,32]. Raman spectroscopy offers high flexibility of excitation wavelengths and sample properties and a higher spatial resolution, often at a higher cost [21,26,27,33]. A more detailed explanation of Raman and IR spectroscopy and their ability to differentiate microorganisms can be found in the literature [21,23,25,28,34,35,36].

The suitability of Raman spectroscopy for the identification of bacteria at the genus, species, and strain level has already been demonstrated [37,38,39,40]. Since many factors and parameters have an influence on the sample and the resulting spectrum, the exact consideration of the influencing factors is an important approach [41]. The use of Raman spectroscopy to study the effect of stress factors on bacteria has typically been limited to specific factors or individual microorganisms. Most Raman spectroscopic studies on how bacteria respond to stress stimuli have used *Escherichia coli* (*E. coli*) as an example. Studies on *E. coli* include the effect of different antibiotics [42,43,44,45], as well as the effect of storage time and sample preparation such as centrifugation [46], the influence of CO_2_ [47], the effect of alcohols [45,48,49], and the effect of temperature and different growth media [50]. In addition, investigations on the different stages of the lifecycle of *E. coli*, *Vibrio vulnificus*, *Pseudomonas aeruginosa*, and *Staphylococcus aureus* [14]; the metabolic monitoring of *Metschnikowia* sp. under different temperatures and C:N ratios [51]; and the effect of UV radiation on *E. coli*, *Serratia marcescens*, and *Micrococcus luteus* (*M. luteus*) [52] have been conducted. Furthermore, the influence of transportation and storage on *E. coli*, *Klebsiella terrigena*, *Listeria innocua*, *Pseudomonas stutzeri*, *Staphylococcus cohnii* and *Staphylococcus warneri* [22]; the spore composition of *Lysinibacillus boronitolerans* in different broth media [53]; and the impact of NaCl, MgSO_4_, and acetate on *Synechocystis* PCC6803 [54] have been investigated. However, to the best knowledge of the authors the investigation of different stress factors and storage times on diverse food relevant microorganisms has not been reported so far in the peer-reviewed literature.

As a result, this study’s goal was to extend the standard models for various defined stress factors on microorganisms that are significant in the food sector (*Bacillus subtilis*; three *E. coli* strains; *M. luteus*; *Brochothrix thermosphacta*; two *Pseudomonas fluorescens* strains; and *Bacillus thuringiensis israelensis*) in order to validate the impact of the stress variables on the quality and reliability of the classification model. In the same context, the spectral variations of different microorganisms caused by stress influence can be noticed, as can attempts to standardize Raman research on biological material in terms of sample preparation and pre-processing.

## 2. Materials and Methods

### 2.1. Bacterial Cultures and Sample Preparation

For this study, the food spoilage-relevant bacteria were cultivated on a nutrient agar with the composition of 18 g/L agar-agar, 10 g/L meat extract, 10 g/L meat peptone and 5 g/L sodium chloride (Merck KGaA, Darmstadt, Germany). The bacteria were as follows:

*Escherichia coli* K12 DSM 498, TOP10, and HB101; *Micrococcus luteus* DSM 20030; *Brochothrix thermosphacta* DSM 20171 (*B. therm*); *Pseudomonas fluorescens* (*Ps. fluor*) DSM 4358 and DSM 50090; *Bacillus subtilis* DSM 10 (*B. sub*); and *Bacillus thuringiensis israelensis* DSM 5724 (*B. tii*). 

The microbial samples to be examined were taken directly from the medium by means of a swab through a stainless steel cylinder without further sample preparation [40,55]. Raman spectra of samples that were cultivated under lifetime stress conditions were recorded immediately after sampling. Otherwise, the samples were subjected to sampling stress and examined spectroscopically without further incubation time after the stress impact.

### 2.2. Sample Treatment

In addition to the regular reference treatment, the microorganisms were exposed to acidic and alkaline incubation, incubation at lower temperature and incubation under 2-propanol influence, which are summarized as lifetime stress conditions. Furthermore, they were subjected to different sampling conditions (heat sampling, cold sampling, and desiccation).

All microorganisms were incubated in a Binder BD 240 (BINDER GmbH, Tuttlingen, Germany) incubator.

#### 2.2.1. Lifetime Stress Conditions

All samples were cultivated according to DSMZ (Leibniz Institut DSMZ, German Collection of Microorganisms and Cell Cultures, Germany) guidelines, except for those that were stressed by incubation at 25 °C. No further sample preparation, like the drying steps of the sample were performed after sampling.

In addition to the reference samples and the samples that were cultivated at 25 °C, the microorganisms were exposed to pH stress. For this, a hydrochloric acid (HCl) solution with pH 1 (36%, Alfa Aesar, USA; confirmed using pH indicator paper, Th. Geyer GmbH & Co. KG, Renningen, Germany) was prepared, as well as a sodium hydroxide solution (sodium hydroxide pellets, Merck, Darmstadt, Germany) with pH 13. The agar plates were thoroughly covered with a 2 mL solution of hydrochloric acid or sodium hydroxide and the inoculation was performed on the covered agar plates.

Analogously to the acidic and alkaline stress incubation, the bacteria were stressed with 2-propanol (99.9%, Höfer Chemie GmbH, Kleinblittersdorf, Germany).

#### 2.2.2. Sampling Stress Conditions

Microorganisms that were exposed to sampling stress conditions were sampled from regular treated samples. For cold sampling they were covered with liquid nitrogen for 60 s and instantly measured. The heat-dried samples were dried for 60 min at 50 °C and instantaneously measured, whereas the desiccated samples were dried for 60 min over silica gel and instantly measured.

### 2.3. Instrumentation

A SENTERRA Raman Microscope (Bruker Optics GmbH, Ettlingen, Germany) with a 785 nm diode laser and a charge-coupled device (CCD) detector was employed in this investigation. The microbiological samples were placed on a motorized XYZ sample stage (Märzhäuser Wetzlar GmbH & Co. KG, Wetzlar, Germany) and focused with a 50× LMPlanFL N objective lens (Olympus K.K, Shinjuku, Tokyo, Japan). The OPUS 7.5 Raman environment software was used for data acquisition and control.

All measurements were performed with a 100 mW initial laser power, an integration time of eight seconds, and ten co-additions. A spectral range of 410–1790 cm^−1^ with a spectral resolution of 3–5 cm^−1^ was chosen to shorten the measuring time.

### 2.4. Data Handling and Statistical Analysis

Raman spectra were truncated to the range of 600–1200 cm^−1^ to cover the most relevant bacterial Raman characteristics and sum normalized (OriginPro 2019b, OriginLab Corporation, Northampton, MA, USA).

**Figure 1:**
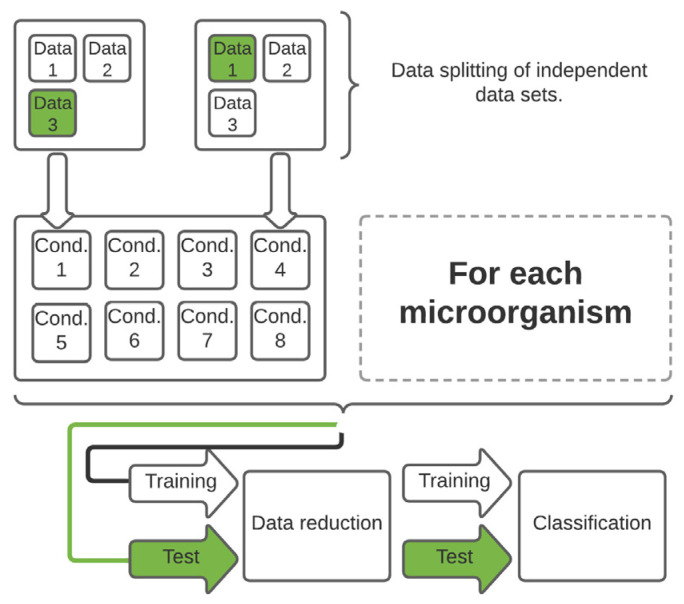
The data splitting, reduction and classification are depicted in this diagram. The corresponding information about the measured microorganisms, stress conditions, and storage times can be found in the training and test data set.

Independent training and test data sets were built so that one of three independent data sets of each stress condition could be used as a test data set (Figure 1). Table S1 in the supplementary material contains detailed information on the exact splitting pattern and the time duration during which bacteria were exposed to lifetime stress conditions.

Using LabVIEW 2016 and OriginPro 2019b, a principal component analysis (PCA), an unsupervised chemometric technique was executed to the training data set to reduce the data set’s dimensionality [56]. The test data sets were converted to the dimensional space of the training data set by applying the training data set’s descriptive statistics and the loadings of the performed PCA to the test data set.

A canonical discriminant analysis (CDA), which uses a linear combination of the data variables to maximize the ratio of between-group and within-group variations of the distinct classes was used in OriginPro 2019b for classification [57,58].

## 3. Results and Discussion

**Figure 2:**
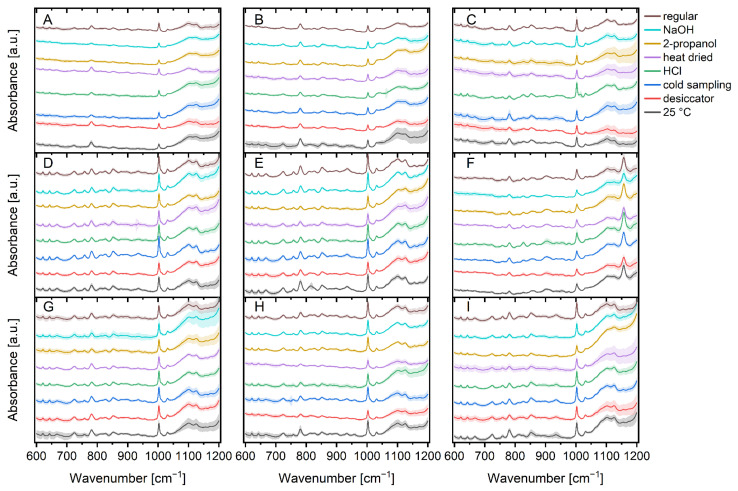
Stacked mean Raman spectra of the normalized data of a total of 5,450 spectra subdivided into seven stress conditions and regular treatment of *B. sub* (**A**), *B. therm* (**B**), *B. tii* (**C**), *E. coli* K12 (**D**), *E. coli* HB101 (**E**), *M. luteus* (**F**), *Ps. fluor* 4358 (**G**), *Ps. fluor* 50090 (**H**) and *E. coli* TOP10 (**I**). Pale colored bands around the mean value represent the standard deviations.

Figure 2 illustrates the mean Raman spectra of bacteria under regular cultivation conditions versus bacteria under different stress conditions, along with their standard deviations.

A closer look at the spectra reveals a few variations between the microorganisms. For example, the region between 650 cm^−1^ and 900 cm^−1^ (proteins, polysaccharides, nucleic acids) [11,25,50,59] which is distinctive for specific bacteria, shows not only differences between Bacilli and *E. coli*, but also variances between Bacilli. For example, the vibration in the cytochrome and DNA [43,50] region around 750 cm^−1^ varies slightly between Bacilli, while the vibration in the region around 890 cm^−1^ (protein, polysaccharide) [11,43] is more pronounced in Bacilli and the vibration in the guanine/DNA region around 665 cm^−1^ [43,60] is less pronounced in the other microorganisms measured. Aside from the obvious difference due to the sarcinaxanthine carotenoid peak at 1157 cm^−1^ [61,62], *M. luteus*, *M. luteus* and *B. therm* also have a band at 1047 cm^−1^ which can be assigned to carbohydrates [43,63] that the other species do not have.

Apart from the differences between the individual microorganisms, there are a few differences between the various stressors of the individual microbial species. The detection of a peak at 1016 cm^−1^ for HCl stressed *B. tii* is by far the most noticeable change. This peak is not found in any other stress factor and can be linked to the symmetric ring breathing vibration of calcium dipicolinic acid during bacterial spore germination [64,65,66,67]. *M. luteus* bacteria cultured at 25 °C possess less exposed tyrosine (828 cm^−1^) [11] than those incubated at other temperatures. Incubation with 2-propanol and sodium hydroxide reveals additional impact on DNA/RNA occurrence in *B. sub* as the peaks for the nucleic acids (810 cm^−1^) [63,68] and bases (665 cm^−1^, 722 cm^−1^, 783 cm^−1^, 828 cm^−1^) [11] are substantially less pronounced. Nucleic acids and tyrosine (810 cm^−1^; 828 cm^−1^) [11,63,68] are also impacted in 25 °C incubated *E. coli* K12 as instead of two peaks, there is only one peak with considerably higher intensity. *E. coli* TOP10 exhibits spectral changes in DNA/RNA and carbohydrates (1100–1130 cm^−1^) [69,70] after incubation with 2-propanol, as well as changed peak ratios for nucleic acids and tyrosine (810 cm^−1^/828 cm^−1^) [11,63,68] ranges after incubation at 25 °C. Such alterations are also found for HCl stressed *Ps. fluor* 50090 and NaOH stressed *Ps. fluor* 4358 in the range of 1100–1130 cm^−1^ (DNA/RNA and carbohydrates) [69,70]. In addition to the increased intensity, there is a shift in favor of carbohydrates [70]. This change can also be seen in HCl and 2-propanol stressed *B. therm.* Furthermore, *B. therm* incubated at 25 °C exhibits more pronounced peaks at 667 cm^−1^ and 810 cm^−1^ (guanine und nucleic acids) [11,63,68] compared to the other parameters.

As spectral variations between specific bacteria and stress conditions are founded on the differences in cell proteins, nucleic acids, lipopolysaccharides, and lipids, visual discrimination of the complete 5450 spectra (Figure 2) is nearly unachievable [10] and chemometric techniques can help with classification.

To achieve optimal model development and avoid overfitting (performance plot: Figure S1), 30 PCs were selected for discriminant analysis model building.

Because there was no substantial equality in the covariance matrices of the training data classes, a quadratic discriminant function was chosen rather than a linear discriminant function [45,46,47].

The classification and cross-validation errors for the training data were 0.78% and 1.5%, respectively. To assess the robustness, accuracy, and reproducibility of the constructed classification model, independent test data sets were added to the model.

**Figure 3:**
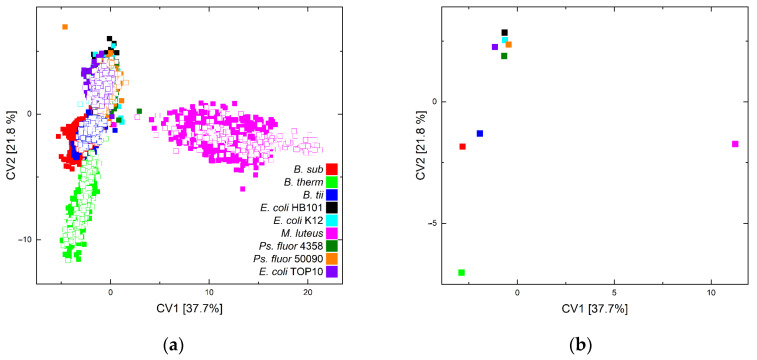
Scatter plots of canonical variable 1 vs. 2 of the QDA of all training data (solid squares) and the independent test data (unfilled squares) of all microorganisms and sampling or lifetime conditions (**a**) and of the group centroids of the training data (**b**).

Figure 3 depicts canonical variables (CV) 1 and 2 of the quadratic discriminant analysis (QDA) of training (solid squares) and test data (unfilled squares) (a) and the group centroids of the training data for CV1 vs. CV2 (b).

The above-mentioned spectral differences are also reflected in the graphical depiction of the discriminant analysis. The reported differences clearly differentiate the point clouds of *M. luteus* and *B. therm* from the other point clouds. However, due to the magnitude of the point clouds, further visual distinction is difficult in the plot. The plot of the group centroids, on the other hand, indicates that *M. luteus* is well separated from the other groups by its obvious extra features and *B. therm* is separated from the other bacteria, although the Bacilli cluster together overall. The separation of *E. coli* and *Ps. fluorescens* between or among themselves does not appear to be achievable.

However, in general, the independent test data are found in the space of the training data, and so the assignment of these data is achievable.

**Figure 4:**
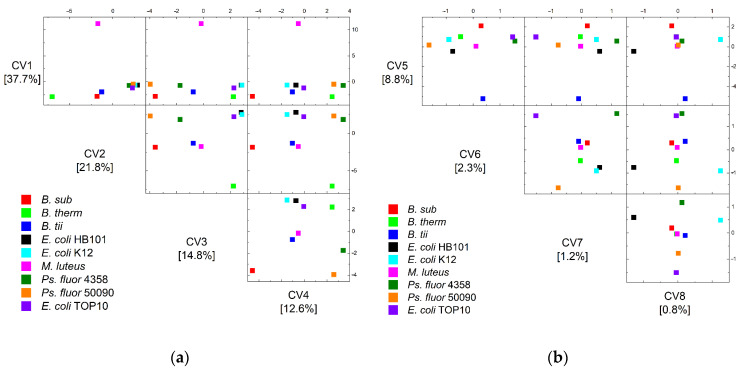
Scatter matrix plots of canonical variable 1–4 (**a**) and canonical variable 5–8 (**b**) of the QDA of the group centroids of the training data of all nine microorganisms and their eight sampling and lifetime conditions.

Figure 4 depicts the group centroids in the eight dimensions of the discriminant analysis in order to analyze the separation of the two remaining bacilli, particularly the separation of *E. coli* and *Ps. fluorescens*.

This demonstrates that all Bacilli are separated in the first three dimensions, but Pseudomonads and *E. coli* require more dimensions in order to be completely separated. The clear distinction of *E. coli* germs in particular is only achievable in the eighth dimension.

The classification results of the independent test data are provided in a confusion matrix (Table 1) which gives the number of spectra categorized to the correct (diagonal) or incorrect predicted class.

The test data categorization and, as a result, the creation of a robust and applicable model was successful. The independent test data classification error rate was 12.5% and was distributed throughout all classes except *M. luteus*.

Table 2 offers a detailed assessment of the classification errors on the sub-class level because the overall dataset is made up of a significant number of sub-datasets per microorganism.

The improper assignment of *E. coli* K12 to TOP10 and HB101 and vice versa, as well as the incorrect classification of *Ps. fluorescens* 50090 to *Ps. fluorescens* 4358 and vice versa, accounted for most of the errors. 

The graphical representation of the classification results already indicated the problem of separation for *Ps. fluorescens*, but especially for *E. coli* separation. The accumulation of 187 misclassifications in the area of *E. coli* out of a total of 232 misclassifications shows that the developed model has good separating power but has weaknesses for separating *E. coli* strains. The modification of the model in such a way that the model for the differentiation of genera, species, and strain level is retained and the differentiation of *E. coli* remains at the species level leads to a false classification rate of only 2.4%.

In comparison to other studies concerning the determination of microorganisms, as well as the influence of stress factors on microorganisms by Raman spectroscopy which partly point to a stricter standardization of the methods [46,71,72] and mainly employ complex and time-consuming sample preparation [44,50,52,73], the presented results show that a reconsideration of the common practice would be helpful, particularly with regard to real-world samples. Because the relevant literature focuses on the analysis of stress factors on specific microorganisms [42,43,44,45,46,48,49,50] or individual stress factors on a few microorganisms [14,52], it was possible to confirm the results observed in this study, namely that the areas influenced by stress factors in microorganisms are primarily found in proteins, lipids, nucleic acids, and polysaccharides [44,45,49,50,52,73]. The combination of numerous stress factors with the traditional reference samples in a model also demonstrated that the classification of unknown samples is not inferior to the classification rates of earlier studies [39,49,74].

In summary, despite the various spectral changes in the range of proteins, lipids, nucleic acids and polysaccharides due to the eight different stress factors applied (lifetime and sampling conditions) to nine different microorganisms, a robust and reliable classification model for food-relevant microorganisms down to strain level could be developed.

## 4. Conclusions

Stress causes alterations in the Raman spectral characteristics of food-relevant microorganisms. Regardless of cultivation conditions, sampling and storage time, a method using simple sample preparation, rapid measurement by Raman microspectroscopy, and chemometrics was developed for the rapid and non-destructive analysis of food-relevant bacteria. A robust and reliable model was created using a canonical discriminant analysis to discriminate nine different microorganisms at genus down to strain level, despite their storage time and sampling of lifetime condition, with about 12% misclassification for independent test data accuracy. The modification of the model for the differentiation on genera, species and strains was retained and the differentiation of *E. coli* remained at the species level, leading to an overall accuracy of 97.6%.

Compared to reference microorganisms that were cultivated under specified standards, stressed microorganisms showed alterations in the spectral range of lipids, nucleic acids, polysaccharides, and proteins.

The results approve the potential of Raman microspectroscopy for the discrimination of bacteria and interpretation of microbial stress responses. Additionally, they indicate that sample preparation and standardization should be reconsidered, and existing standardized databases should also contain stress conditions.

## Figures and Tables

**Table 1:** Confusion matrix for the independent test data set: Rows represent the observed groups and columns represent the expected groups. The values in the table’s diagonal correspond to the correct grouping of observations.

	Predicted Class								
Class	*B. sub*	*B. therm*	*B. tii*	*E. coli.* HB101	*E. coli* K12	*M. luteus*	*Ps. fluor* 4358	*Ps. fluor* 50090	*E. coli* TOP10
*B. sub*	**199**	0	1	0	0	0	0	0	0
*B. therm*	0	**204**	1	0	0	0	0	0	0
*B. tii*	0	0	**199**	0	0	0	0	0	1
*E. coli* HB101	0	0	0	**138**	62	0	0	0	5
*E. coli* K12	0	0	0	24	**139**	0	0	0	42
*M. luteus*	0	0	0	0	0	**210**	0	0	0
*Ps. fluor* 4358	0	0	0	7	0	0	**197**	6	0
*Ps. fluor* 50090	0	0	0	0	0	0	29	**181**	0
*E. coli* TOP10	0	0	0	3	51	0	0	0	**151**

**Table 2:** Examination of the classification mistakes in the independent test data set at the sub-dataset level. The figures represent the overall number of misclassified spectra in the relevant sub-dataset.

Error Distribution	*B. tii*	*E. coli* HB101	*E. coli* K12	*Ps. fluor* 4358	*Ps. fluor* 50090	*E. coli* TOP10
*B. sub*—cold sampling	**1**					
*B. therm*—25 °C	**1**					
*B. tii*—heat dried						**1**
*E. coli* HB101—desiccator			**4**			
*E. coli* HB101—cold sampling			**1**			**3**
*E. coli* HB101—HCl			**22**			**1**
*E. coli* HB101—heat dried			**1**			
*E. coli* HB101—2-propanol			**10**			**1**
*E. coli* HB101—NaOH			**18**			
*E. coli* HB101—regular			**6**			
*E. coli* K12—desiccator						**5**
*E. coli* K12—HCl		**3**				**9**
*E. coli* K12—heat dried		**1**				**20**
*E. coli* K12—2-propanol		**4**				**1**
*E. coli* K12—NaOH		**16**				**7**
*Ps. fluor* 4358—desiccator		**7**			**6**	
*Ps. fluor* 50090—25 °C				**3**		
*Ps. fluor* 50090—HCl				**22**		
*Ps. fluor* 50090—heat dried				**4**		
*E. coli* TOP10—25 °C		**2**	**15**			
*E. coli* TOP10—desiccator			**12**			
*E. coli* TOP10—heat dried			**1**			
*E. coli* TOP10—2-propanol			**1**			
*E. coli* TOP10—regular		**1**	**22**			

## Data Availability

The data presented in this study are available on request from the corresponding author.

## Supplementary Materials

The following are available online at https://www.mdpi.com/article/10.3390/foods11101506/s1, Table S1: detailed information of data splitting; Figure S1: check for overfitting.
