# Supplementary material for: Discrimination of Stressed and Non-Stressed Food-Related Bacteria Using Raman-Microspectroscopy
Source: Foods. 2022 May 22;11(10):1506. doi: 10.3390/foods11101506 (PMC9141442; doi:10.3390/foods11101506)
Supplement: Supplementary file 1 [file foods-11-01506-s001.zip › foods-1686852-supplementary.pdf]

Supplementary material

Table 1. Scheme for data splitting for trained and tested microorganisms. Each data set's storage time is specified in days. Test data are highlighted in green.

	25 °C			Dessicator			Cold sampling			HCl			Heat dried			2-propanol			NaOH			Regular		
**Data set**	**1**	**2**	**3**	**1**	**2**	**3**	**1**	**2**	**3**	**1**	**2**	**3**	**1**	**2**	**3**	**1**	**2**	**3**	**1**	**2**	**3**	**1**	**2**	**3**
B. sub	1	2	3	8	9	10	8	12	14	3	9	14	7	9	10	1	9	15	1	6	7	4	6	8
B. therm	4	9	1	9	15	1	3	7	20	9	10	14	8	14	16	10	11	15	2	7	8	5	7	15
B. tii	1	9	1	8	9	10	11	12	3	7	8	9	7	9	10	2	8	15	6	7	8	5	6	8
***E. coli* K12**	4	9	1	9	15	1	3	7	20	6	9	14	8	14	16	2	9	15	2	7	8	21	5	12
***E. coli* HB101**	11	9	1	9	10	1	3	15	7	6	8	14	8	9	11	2	9	16	3	6	7	21	1	7
M. luteus	8	2	13	18	10	16	3	11	15	3	9	14	8	9	11	1	9	15	1	6	7	21	18	7
***Ps. fluor* 4358**	1	8	1	9	15	1	3	16	20	6	8	14	8	14	16	2	9	15	6	6	7	12	5	18
***Ps. fluor* 50090**	8	4	1	9	15	1	3	11	23	6	9	14	8	14	16	1	8	16	6	6	7	5	21	12
***E. coli* TOP10**	1	9	1	15	15	1	16	20	7	8	9	14	17	14	16	2	8	15	2	7	8	1	5	12

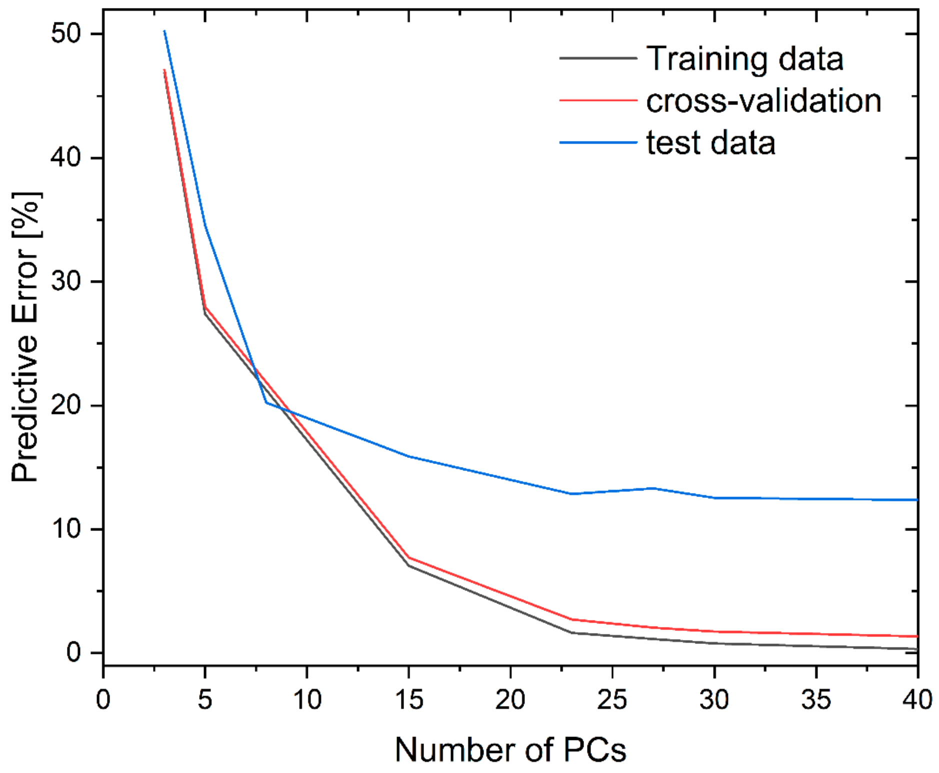

Figure S1. Check for Overfitting.
